# Preliminary evaluation of full volume strain measurement in patellar cartilage following osteochondral allograft transplantation using magnetic resonance imaging

**DOI:** 10.3389/fbioe.2025.1701592

**Published:** 2026-01-07

**Authors:** Michael A. Hernández Lamberty, Carla Nathaly Villacís Núñez, Ulrich Scheven, John A. Grant, Ellen M. Arruda, Rhima M. Coleman

**Affiliations:** 1 Department of Mechanical Engineering, University of Michigan, Ann Arbor, MI, United States; 2 Department of Orthopaedic Surgery, University of Michigan, Ann Arbor, MI, United States; 3 Department of Biomedical Engineering, University of Michigan, Ann Arbor, MI, United States; 4 Program in Macromolecular Science and Engineering, University of Michigan, Ann Arbor, MI, United States

**Keywords:** biomechanical imaging, cartilage, magnetic resonance imaging (MRI), osteochondral allograft transplant, patella, injuries

## Abstract

**Introduction:**

Articular cartilage (AC) defects of the patellofemoral joint (PFJ) are clinically challenging and mechanically demanding. Osteochondral allograft (OCA) transplantation is the standard treatment for large cartilage injuries; however, little is known about intra-tissue mechanics after transplantation. Computational models suggest that cartilage thickness mismatch concentrates stresses at donor–recipient interfaces in OCA-treated patella, but direct experimental evidence is scarce. Local cartilage strain is closely linked to tissue health; therefore, the goal of this work was to provide a preliminary, full volume assessment of patellar cartilage mechanics before and after OCA transplantation.

**Methods:**

A displacement-encoded MRI sequence was used to quantify full volume displacement and strain fields in human patellar AC before and after OCA transplantation under controlled indentation. Intact cadaveric patellae (n = 4) were prepared, with three serving as recipients and one as donor. Samples were cyclically compressed in a custom-built rig using nominal displacements of 1 and 2 mm. The complex phase data were unwrapped and converted to displacements; the Green–Lagrange strain tensor was computed using a finite element framework in FEniCS. Minimum principal strain ( $E_{\min}$ ) and maximum shear strain ( $E_{maxshear}$ ) were analyzed. Donor–recipient step-off distance, representing cartilage-level geometric mismatch, was measured at the graft interface.

**Results:**

Global displacement fields were similar between intact and OCA samples, with spherical indentation exhibiting through-thickness compression and lateral displacement in longitudinal and transverse directions. $E_{\min}$ localized beneath the indenter, while $E_{maxshear}$ concentrated near the articular surface. OCA-transplanted samples exhibited localized changes in strain distribution near portions of the graft rim, though these features varied across samples. Top-view percentile maps highlighted redistributed high-strain regions in some OCA samples. Exploratory step-off plots showed sample-specific directional trends between geometric mismatch and donor-recipient strain differences, though these trends were not consistent across all samples.

**Discussion:**

This exploratory study provides the first experimental full volume displacement and strain distributions of patellar cartilage after OCA transplantation. The localized strain variations observed after transplantation should be interpreted descriptively, given the single-donor design and sub-physiological loading. These results establish an experimental foundation for validating computational models of the donor-recipient cartilage interaction and geometric mismatch following OCA transplantation and work investigating OCA mechanics under physiological loading.

## 1 Introduction

Articular cartilage (AC) is a soft tissue found in diarthrodial joints, which provides near-frictionless contact and load transfer between bones (Fox et al., 2009). Its aneural and avascular nature limits its ability to regenerate after injury. Cartilage injuries are common, present in approximately 66% of patients undergoing knee arthroscopy procedures (Årøen et al., 2004; Curl et al., 1997). These defects compromise joint function, contribute to joint pain, and, if untreated, may accelerate the onset of osteoarthritis (Bhosale and Richardson, 2008).

The patellofemoral joint (PFJ) is particularly vulnerable to cartilage damage because of its unique anatomy and high mechanical demands. Chondral lesions in the PFJ account for up to 44% of cartilage defects in the knee (Widuchowski et al., 2007; Hjelle et al., 2002). Among athletes, 18%–27% of knee cartilage injuries are localized at this joint (Flanigan et al., 2010). PFJ lesions may result from recurrent patellar dislocation, traumatic impact, or cartilage degeneration (Brophy et al., 2017). When they develop, these defects are exposed to contact forces that can reach 6.5 times body weight (BW), amplifying tissue damage. Abnormal patellar tracking, trochlear or patellar dysplasia, and altered limb alignment further disrupt load distribution, focusing stresses on the compromised cartilage regions (Jibri et al., 2019). Additionally, variability in PFJ morphology and patient-specific heterogeneity may confound efforts to restore articular cartilage.

Several surgical methods exist to address full-thickness injuries in the AC, such as autologous chondrocyte implantation (ACI), osteochondral autograft transfer (OAT), and osteochondral allograft (OCA) transplantation (Brophy et al., 2017; Mestriner et al., 2018; Andrade et al., 2021), depending on defect size. OCA transplantation is the surgical standard for addressing large (> $2\;{cm}^{2}$ ) full-thickness cartilage injuries (Hevesi et al., 2021) and has two major advantages compared to other cartilage restoration procedures. First, OCA transplants are performed in a single-stage surgery in which the mature hyaline articular cartilage is press-fit to the prepared site, and the cartilage surface is restored. This fixation method enables bone-to-bone healing, which increases the potential for faster rehabilitation. Second, OCA procedures have demonstrated excellent patient-reported outcomes and high rates of return to sport (Crawford et al., 2019). OCA transplant procedures are also a potential option for bipolar lesions in the PFJ (Gracitelli et al., 2015a) and for patients with post-traumatic osteochondral defects that are too young for arthroplasty (Gracitelli et al., 2015b). Despite these advantages, the limited availability of osteochondral allografts continues to be one of the major disadvantages of the procedure. (Haikal et al., 2023). Even when grafts are available, chondrocyte viability may decline during storage prior to surgery (Cook et al., 2016; Pallante et al., 2009). OCA transplant procedures in the knee have reported survival rates of 86.7%, 78.7%, and 72.8% at 5, 10, and 15 years, respectively (Familiari et al., 2018). However, beyond 15 years, OCA procedures in the patella are associated with a 20.1% failure rate and a 51.6% reoperation rate (Chahla et al., 2019). These outcomes suggest that unaddressed mechanical factors in the PFJ may contribute to graft deterioration. However, research focused on the intra-joint mechanics following OCA transplantation is scarce, especially in the patella.

Articular cartilage congruity and osseous graft integration are the primary parameters used to evaluate OCA transplant success (Lai et al., 2022). While factors such as proud grafts and OCA graft thickness (including both the articular cartilage and the attached bone) have been studied (Ackermann et al., 2019; Pallante et al., 2012; Murphy et al., 2014), matching the transplant cartilage thickness to that of the patient is not the standard protocol. Using finite element analysis, we have previously shown that a donor-to-recipient (D/R) cartilage thickness disparity in the patella after an OCA procedure can lead to stress levels up to double the applied stress in the graft boundary (Rosario et al., 2023). The high stress regions (HSR) caused by this disparity could precipitate cell death at the boundary of the graft, extracellular matrix damage, or poor graft integration in the transplanted site (Bonnevie et al., 2018; Kosonen et al., 2023). The generated HSR may explain one of the potential factors that decrease the survivability of OCA transplants in the patella. This highlights the need to describe the strain distribution in the patella after an OCA transplant procedure using both experimental and computational methods.

Full field material characterization methods can be used to experimentally determine strain maps during mechanical testing. Digital image correlation (DIC) (Luetkemeyer et al., 2021; Chu et al., 1985) is one of the main strain measurement methods; however, it is limited to probing the surface of the material, assuming in-plane deformation. For complex three-dimensional tissues like the articular cartilage of the patella, in-plane deformation assumptions limit our understanding of internal mechanics.

Displacement-encoded magnetic resonance imaging (MRI) (Aletras et al., 1999) on the other hand, uses complex-valued phase data to determine full volume displacement variation between deformed and reference configurations, which would enable the characterization of the compression biomechanics of the articular cartilage. We recently developed a custom displacement-encoded MRI sequence, termed alternating pulse field gradient stimulated echo imaging (APGSTEi) (Scheven et al., 2020), with which the anterior cruciate ligaments and patellar tendon were modeled (Luetkemeyer et al., 2021), and tear growth mechanisms of rotator cuff tendon tears were characterized (Villacís Núñez et al., 2025).

In this work, we applied the APGSTEi sequence to demonstrate the feasibility of obtaining full volume, three-dimensional displacement and strain maps of human patellar cartilage under controlled compressions. This approach overcomes the limitations of surface-based methods by providing full volume data at voxel-level resolution. Applying this technique to both intact and OCA-treated patellar samples allowed us to visualize how strain is distributed through the thickness of the cartilage, including at the donor-recipient interface, where FE simulations have predicted elevated stress and pathways of potential failure of the OCA transplant procedure (Rosario et al., 2023). This study focuses on the methodological capability of APGSTEi to capture intratissue strain patterns in the patella cartilage and explore how geometric features such as graft congruity may correspond to local mechanical response. This data and methods establish an experimental foundation for future work evaluating OCA mechanics under physiological loading and for validating computational models of the donor-recipient cartilage interaction.

## 2 Materials and methods

### 2.1 Experimentation

To characterize the mechanical response of the patella articular cartilage, our procedure involved analyzing full volume displacement and strain before and after performing an OCA transplant procedure, using the APGSTEi method. Loading and MRI protocols were previously developed for the constitutive modeling of the anterior cruciate ligament and analysis of rotator cuff tendons (Luetkemeyer et al., 2021; Villacís Núñez et al., 2025; Estrada et al., 2020). The sample preparation, loading protocol, OCA transplant procedure, and MRI sequence are described below.

#### 2.1.1 Sample preparation

**FIGURE 1:**
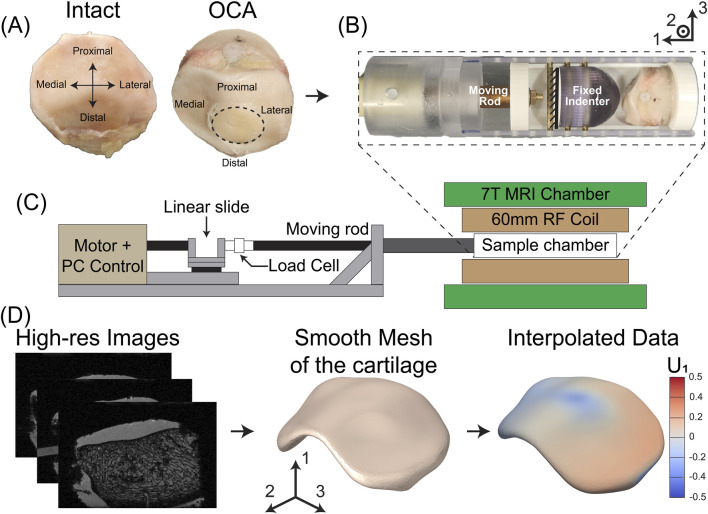
Experimental setup and workflow for patella testing and analysis. **(A)** Representative image of intact patella sample (left) and the same sample after OCA transplantation (right). **(B)** Compression assembly with a fixed indenter and a translating fixture containing the patella sample, driven by a motor, which applied controlled displacements to the patella surface. **(C)** MRI setup: the compression assembly from **(B)** was placed inside a 60 mm RF coil and imaged within a 7T MRI chamber. **(D)** Image processing workflow: high-resolution MRI images are segmented to create binary masks, which were reconstructed into 3D cartilage volumes. These masks underwent remeshing and smoothing to be used for interpolation of displacement and strain data.

Intact human patellae from left knees were obtained for this study (n = 4). These samples were provided by James Ashton Miller’s team at the University of Michigan. Table 1 contains information for the samples used for this study. The use and processing of these cadaveric samples were approved by the University of Michigan’s Institutional Biosafety Committee. Samples were removed from the freezer (−20 °C) and thawed overnight at 4 °C. Three 6.35 mm diameter screws were cored out and filled with Dragon Skin™ 20 (Smooth-On). These screws were placed into holes that were made in the base (proximal side) (Figure 1A), medial, and lateral sides of the patella to facilitate image registration between intact and OCA-transplanted samples. The anterior side of the patella (bone side) was fixed in poly (methyl methacrylate) (PMMA). After the PMMA hardened, a cylindrical core of the patella-PMMA cast assembly was extracted using a 44.45 mm internal diameter hole saw (Diablo Tools). The PMMA core provided a flat surface that consistently adhered to the translating fixture to load the patella (Figure 1B), conserving alignment during each test. The articular cartilage surface of the patella was hydrated in 1X phosphate-buffered saline (Thermo Fisher Scientific, Inc., Waltham, MA) for a minimum of 6 h before testing.

**TABLE 1:** Characteristics of the samples used for the experiments.

Sample characteristics	Mean ± SD
Age [years]	36.3 ± 12.0
Weight [kg]	60.2 ± 6.9
Height [cm]	175.9 ± 4.5
Cartilage thickness [mm]	3.9 ± 0.4
1 mm displacement force (intact) [N]	61 ± 12
1 mm displacement force (OCA) [N]	58 ± 3
2 mm displacement force (intact) [N]	120 ± 27
2 mm displacement force (OCA) [N]	91 ± 5

#### 2.1.2 Loading protocol

Samples were mounted in MRI-compatible 3D printed fixtures (Figure 1B) using cyanoacrylate and brass nuts. To ensure the patellar samples were consistently compressed in the same location, the sample’s proximal side was aligned with the 2-direction in the global axis (Figure 1B). This assembly was placed inside a polycarbonate cylindrical chamber designed in-house. A 25.4 mm indenter was fixed to the chamber to compress the surface of the intact patella (Figure 1B), and the entire assembly was attached to the moving rod. The displacement of the moving rod in the mechanical testing rig was controlled and applied using a computer-driven linear stepper motor (L5918S2008-T1-X2-A50; Nanotec Electronic GmbH and KG, Germany) in series with a load cell (LCM300; Futek Advanced Sensor Technology Inc., Irvine, CA, United States). The stepped linear actuator in our design can provide forces up to 400 N. To prevent sample dehydration during testing, the sample chamber was sealed with a cap and taped around all edges using waterproof tape. This was overlayed with a heat-shrinking bag that was clamped to a protective tube covering the moving rod. The sample chamber was placed at the center of a 60 mm radiofrequency coil (RF) (Agilent Millipede), inside a 7 T MRI machine (Agilent) (Figure 1C). Before each set of tests was performed, a reference position was determined using a force threshold of 15 N, indicating the start of compression of the cartilage. This was followed by a 10-min pre-conditioning step of 1 mm displacement at 0.33 Hz. Samples were tested using a load-unload cycle synchronized to the APGSTEi sequence (Scheven et al., 2020), as described below.

The displacement and strains of each patella were evaluated in two different cyclical compression protocols. The fixture that contained the patella sample was displaced at 1 mm and 2 mm into the indenter to engage the articular cartilage surface. Peak forces during intact patella compression tests reached 61 ± 12 N (mean ± std dev) and 120 ± 27 N for the 1 mm and 2 mm elongations, respectively. In OCA transplant compression, peak forces were 58 ± 3 N and 91 ± 4 N for the 1 mm and 2 mm stretches (Table 1). Although these forces did not achieve the values observed *in vivo*, which tend to be greater than 0.9x BW, this method yielded comparative data on the strain and deformation field before and after an OCA procedure in a controlled way. This method also provided insight into the accuracy of the graft in replicating the native biomechanics observed in the intact patellae samples.

Due to the compliance of the loading assembly, the applied displacements of 1 mm and 2 mm resulted in cartilage compressions of 0.25 mm and 0.50 mm, respectively at the cartilage surface. We will be referring to the effective elongations as 0.25 mm and 0.50 mm from here on.

#### 2.1.3 APGSTEi protocol

The APGSTEi protocol (Scheven et al., 2020) captures complex-valued images in which the voxel-wise phase is directly proportional to the voxel’s displacement and inversely proportional to the chosen encoding wavelength, λ . This wavelength is an adjustable experimental parameter that determines how sensitively the MRI phase responds to tissue displacements. Short encoding wavelengths increase sensitivity to small displacement but can lead to more phase wrapping and lower signal-to-noise ratio (SNR). In contrast, longer wavelengths reduce sensitivity but offer better SNR and are less prone to phase wrapping. When unwrapped, the phase maps represent the full volume displacement variation between the reference and the deformed (compressed) configuration. The phase images were captured on a low-resolution grid of 96 × 32 × 24 voxels with voxel dimension of 0.33 mm (thickness direction, axis 1) × 1.5 mm (longitudinal direction, axis 2) × 2.0 mm (transverse direction, axis 3). An encoding wavelength of 1 mm was used for the nominal compression sequences of 1 mm and 2 mm. The APGSTEi acquisition of displacement encoded MRI data required about 13 min, for each of the three displacement encoding directions. This corresponds to 192 acquisitions per encoding direction, with a repeat time TR = 3 s. Average displacements rate were set by the time between positional encoding and decoding, set to less than 0.4 s, and the maximal amplitude of displacements, set to 2 mm. Additionally, a high-resolution dataset was acquired prior to each loading sequence using a 3D gradient echo sequence to aid in post-processing steps such as masking and interpolation. This high-resolution dataset was acquired at a resolution of 196 × 128 × 128 voxels and voxel sizes of 0.17 mm × 0.38 mm × 0.38 mm (Figure 1D).

#### 2.1.4 OCA transplant protocol

After sample imaging and loading, the patellae were assigned to donor or recipient roles for the OCA procedure. One specimen (Sample 2) was designated as the donor patella, and the remaining three samples (Samples 1, 3, and 4) served as OCA recipients. Similar to the intact sample preparation, each sample was placed in a PMMA cast for easier handling while creating the OCA defect in the recipient or removing the graft from the donor sample. A patella core was removed from the donor sample using a 19.05 mm internal diameter hole saw (Diablo Tools). The core was harvested from the central region of the articular surface on the posterior aspect of the patella. The center was identified by measuring the width (medial to lateral distance) and height (proximal to distal distance) of the articular cartilage. A clock-face orientation system (3, 6, 9, and 12 o’clock positions) was used to identify the medial, distal, lateral, and proximal sections, respectively, of the core. This plug was then trimmed down to a height of approximately 8 mm (Dekker et al., 2019; Dean et al., 2016).

The three recipient samples were cored to a depth of ∼8 mm to match the height of the donor core. The central location of the recipient core was found using a similar procedure to that used for the donor core. The recipient site was created using a 17.46 mm diameter spade bit, which also produced a small posterior exit hole that facilitated the removal of the donor plug. If the depth of the hole exceeded the height of the donor patella core, small amounts of PMMA were used to fill in the hole and secure the core. A similar clock-face orientation system was made in the recipient such that the donor core aligned with the medial, distal, lateral, and proximal regions of the recipient. The donor core was inserted manually and seated using light axial pressure to achieve a secure press-fit between the donor and recipient, consistent with clinical OCA technique.

**FIGURE 2:**
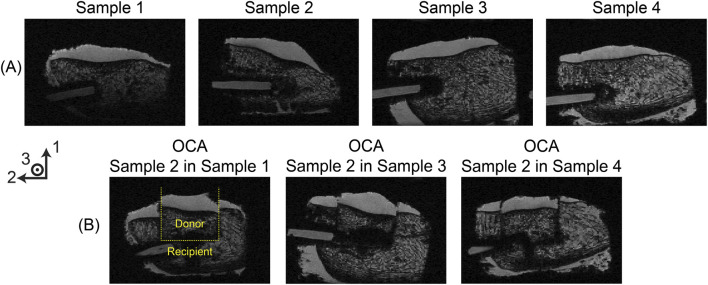
High-resolution MRI images in the 1-2 plane. **(A)** Intact patellae samples, **(B)** the same samples after the OCA transplantation. The first image shows the boundary between the donor graft and the recipient cartilage for reference.

After successfully performing the OCA transplant, the patella was removed from the PMMA using the 44.45 mm internal diameter hole saw. The removed patella and PMMA were then placed on the 3D printed fixture using cyanoacrylate and set up for the loading and MRI protocol previously described. The sample was left to rehydrate in 1X phosphate-buffered saline for a minimum of 6 h prior to mechanical testing. This process was repeated for the other recipient samples using the same donor sample. The samples will be referred to as OCA Sample 1 (Recipient: Sample 1, Donor: Sample 2), OCA Sample 3 (Recipient: Sample 3, Donor: Sample 2), and OCA Sample 4 (Recipient: Sample 4, Donor: Sample 2). Figure 2 contains representative high-resolution images of the intact (Figure 2A) and OCA-treated (Figure 2B) samples.

### 2.2 Data processing

The data processing methods used for the analysis of the complex images obtained from the loading and MRI protocol was previously developed for the analysis of rotator cuff tendon dataset (Villacís Núñez et al., 2025). The procedure is described briefly in the following sections.

#### 2.2.1 Phase unwrapping

Custom built MATLAB scripts (The MathWorks, Inc., version 2024a) were used to preprocess MRI data. The high-resolution scans were used to generate masks of our region of interest by isolating the articular cartilage from the surrounding bone and void space. Individual masks for each sample were created at their reference positions. These masks were dilated using a radius of 3 pixels to capture peripheral voxels with partial-volume displacement information near the boundary of the cartilage.

Displacement-encoded complex images were smoothed using a 3D Gaussian kernel to reduce noise before phase unwrapping. Phase unwrapping was performed using an open-source algorithm from Maier et al., 2015; Maier et al., 2015). The unwrapped phase maps were then converted to real-valued displacements in the native low-resolution grid before further smoothing was performed. The data was upsampled into the high-resolution grid. Using the finite element framework in FEniCS, the spatial gradients of the displacement fields were evaluated from the derivative of the basis functions, and the full Green-Lagrange strain tensor was obtained through the $L^{2}$ projection of this tensor field onto the finite-element space. The eigenvalues of the strain tensor were subsequently calculated to analyze the strain outcomes of interest: the minimum principal strain ( $E_{\min}$ ) and maximum shear strain ( $E_{\max\;shear}$ ); and their directions. $E_{\min}$ corresponds to the most negative eigenvalue of the strain tensor and reflects local compressive strains, which are particularly relevant for cartilage mechanics during loading. The $E_{\max\;shear}$ was calculated as half of the difference between the first and third eigenvalues of the strain tensor, representing the tissue deformation under shear. Quantifying maximum shear strain is important, as shear has been linked to the initiation of apoptosis and cell death in the cartilage (Bonnevie et al., 2018).

#### 2.2.2 Data interpolation and visualization

The masks obtained from the high-resolution images in MATLAB were exported as STL files for further refinement and smoothing. This process was performed in Meshmixer (Autodesk Inc., version 3.5.474) while the meshing of the STL was completed in Hypermesh (Altair Engineering Inc., version 2021). Since STL files only produce surface information, the surfaces were automatically tessellated into triangular elements, with an element size of approximately 0.30 mm. Element quality of the surface triangular mesh was evaluated using standard criteria, including aspect ratio, warpage, skewness, and minimum/maximum interior angles to ensure adequate mesh quality prior to volumetric tetrahedralization. This surface mesh was then converted to a tetrahedral volumetric mesh. This volumetric mesh was used to interpolate the displacement and strain fields using a custom MATLAB script. These values and their corresponding meshes were exported to XDMF format using a Python script (The Python Software Foundation, version 3.12.10), using the FEniCS package and incorporating Dolfin and UFL libraries (Baratta et al., 2023; Alnæs et al., 2014). Visualization of displacement and strain distributions throughout the cartilage volume was performed in Paraview (Sandia National Laboratories, Kitware Inc., Los Alamos National Laboratory, version 5.13.2).

**FIGURE 3:**
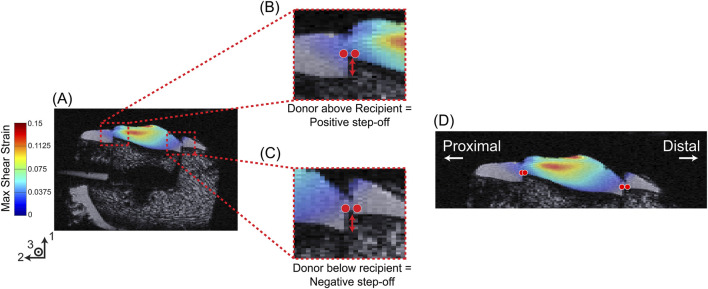
Step-off distance measurement and strain outcome workflow. **(A)** Representative image of the OCA sample with overlayed max shear strain in the 1–2 plane. Representation of a positive **(B)** and negative **(C)** step-off distance measurement. **(D)** Representation of queried locations for the magnitude of strain outcome in both the proximal and distal sides of the recipient and donor cartilage.

Following the strain interpolation, the spatial distribution of the strain outcomes were projected into a top view (2–3 plane) using a column-wise 95th percentile along the through-thickness (1-direction) axis (Figure 6). In this approach, each vertical column of voxels through the cartilage thickness is reduced to a single representative value corresponding to the 95th percentile, which emphasizes the higher strain value within the column while suppressing noise. This method yielded maps that highlighted high-strain regions beneath the indenter and around the graft. This was followed by measuring a “step-off” distance for all samples that contained an OCA transplant. Step-off distance was defined as the difference (in mm) from the start of the subchondral bone of the recipient cartilage and the start of the subchondral bone of the donor graft at the interface. For each slice in the 1–2 plane through the graft, step-off values were sampled at anatomically matched proximal and distal interface locations, producing two complementary “half-circles” arcs that together capture the full circumferential geometry of the graft interface. The distance was measured in voxels using the high-resolution anatomical images, then converted to millimeters based on the voxel dimensions, yielding an accuracy of approximately 0.2 mm. Negative step-off values indicated that, within the queried slice of the 1–2 plane, the donor subchondral bone was recessed relative to the recipient’s (Figure 3C), whereas positive values indicated that the donor subchondral bone was elevated (Figure 3B).

Two strain outcomes of interest were selected to be visualized and compared between samples: $E_{\min}$ and $E_{\max\;shear}$ . The magnitude of the strain (Figure 3A) outcomes was queried initially at the recipient side of the graft interface, and then the same approximate lateral location was queried on the donor side (Figure 3D). The percentage change in strain outcomes from recipient to donor was calculated as:

$$\%\;change\;in\;strain\;from\;recipient\;to\;donor=\frac{{strain}_{\;donor}-{strain}_{\;recipient}}{{strain\;}_{recipient}}\times 100$$

To visualize how geometric mismatch might relate to strain differences, we generated scatter plots with step-off distance as the independent variable and percentage change in $E_{\min}$ and $E_{\max\;shear}$ as the dependent variables (Figure 7). These plots were used strictly as descriptive, exploratory visualizations to observe qualitative trends. Given the limited sample size and the use of a single donor for all three OCA procedures, no formal statistical inference was performed.

## 3 Results

### 3.1 Compressive response of articular cartilage during indentation

The thickness of the patellar cartilage samples was measured to be 3.9 ± 0.4 mm at the location of indentation. Mean forces were lower in OCA-transplanted patellae samples compared to intact samples; however, the limited sample size and donor–recipient pairings precluded statistical inference. At an effective displacement of 0.25 mm (nominal 1 mm), which was 6.4% ± 0.8% compression of the cartilage thickness, intact samples reached 61 ± 12 N, while the OCA samples averaged 58 ± 3 N. At a 0.5 mm displacement (nominal 2 mm), which represented 12.9% ± 1.6% compression of the cartilage thickness, intact samples reached 120 ± 27 N, while OCA samples reached 91 ± 4 N (Table 1).

### 3.2 Full volume displacement maps following indentation

**FIGURE 4:**
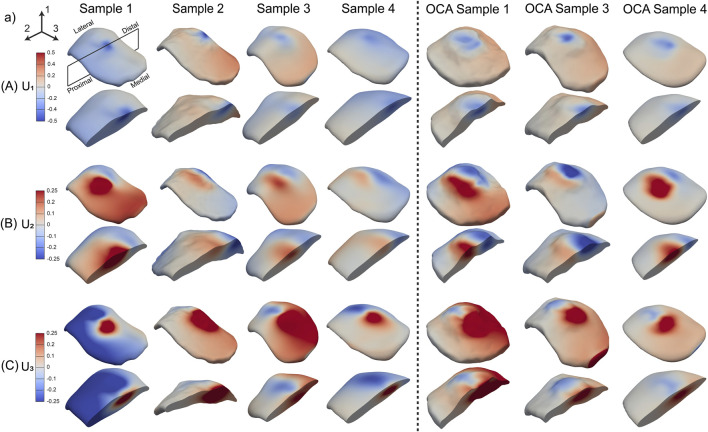
Full volume displacement maps of patellar articular cartilage for an effective displacement of 0.5 mm. **(A)** Through-thickness ( $\left.U_{1}\right)$ , **(B)** longitudinal ( $U_{2}$ , along the proximal-distal axis), and **(C)** transverse ( $U_{3}$ , along the medial-lateral axis) displacements. The longitudinal direction corresponds to the loading direction of the indenter. Columns 1–4 show intact patellae, while columns 5–7 show OCA-treated patellae. The first row of each frame shows the full volume of the patella, while the second row shows a cross-section in the 1–2 plane, through the mid-plane of the indenter.

Based on these compression tests, full volume displacements maps were generated for all samples. Only the 0.50 mm loading condition is shown (Figure 4). In the through-thickness direction ( $U_{1}$ ), spherical contours were observed, which were expected for a spherical indentation (Figure 4A). The largest displacement was observed directly below the indentation location. Displacement fields in the longitudinal ( $U_{2}$ ) and transverse ( $U_{3}$ ) direction were more heterogenous. The longitudinal displacements (Figure 4B) demonstrated lateral movement of the cartilage in opposite directions. This behavior was also observed in the transverse direction (Figure 4C) where the cartilage had lateral movements along the 3-axis. In the OCA samples (Figure 4, last three columns), localized variations in the displacements were observed in all three directions, but the overall displacement behavior was similar to that of the intact samples.

### 3.3 Strain field redistribution occurred after OCA transplantation with rim effects near the grafts

**FIGURE 5:**
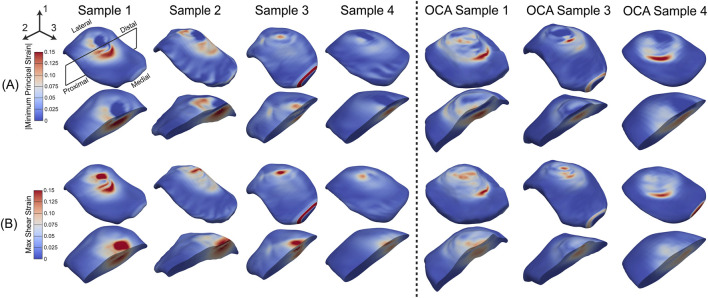
Full volume strain maps of patella articular cartilage for an effective displacement of 0.5 mm. **(A)** Absolute minimum principal strains ( $\left|E_{\min}\right|$ ) and **(B)** maximum shear strains ( $E_{\max\;shear}$ ). Columns 1–4 show the intact patellae samples, while columns 5–7 show the OCA-treated patellae samples. Red regions indicate areas of high compressive or shear strain, while blue regions indicate low strain.

The minimum principal strain (Figure 5A) was localized beneath the indenter, corresponding to the region of highest compressive deformation in the cartilage. The maximum shear strain ( $E_{\max\;shear}$ ) represented the tissue shear deformation generated by the cartilage compression and outward expansion during indentation. The maximum shear strain (Figure 5B) exhibited distributions of strain on the top of the cartilage surface with some shear occurring through the thickness of the cartilage. Among the intact samples, the highest strain intensities were generally observed beneath the indentation, with sample 1 showing the largest magnitudes (Figure 5, column 1). In the OCA-treated samples, overall strain patterns resembled intact samples, but occasional rim effects were evident in some cases. Specifically, OCA samples 1 and 4 displayed bands of elevated minimum principal strain and shear strain adjacent to portions of the graft rim, particularly along the medial edge.

**FIGURE 6:**
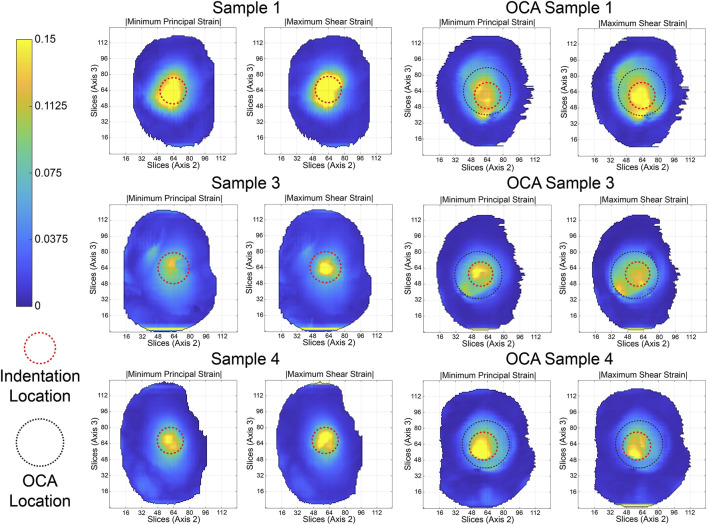
95th percentile strain distributions projected onto a top view of the patella cartilage for an effective displacement of 0.5 mm. Strains are shown in the 2–3 plane. For each sample, adjacent panels display the magnitude of the minimum principal strain ( $E_{\min}$ , left) and maximum shear strain ( $E_{\max\;shear}$ , right) before (first two columns) and after (last two columns) OCA transplantation. Red dashed outlines indicate the indentation site, while black dashed outlines indicate the location of the OCA graft.

In the intact patellae samples, elevated strains were largely confined to the indenter footprint (Figure 6, first two columns), with Sample 1 exhibiting the highest peak intensities. After OCA transplantation, the projected maps showed a larger redistribution of strain around the whole donor cartilage. OCA Samples 1 and 3 displayed elevated strains near the graft perimeter. These patterns illustrate localized changes in strain distribution after transplantation, although the magnitude and extent of the features varied across samples.

### 3.4 Relationship between step-off distance and strain changes in donor-recipient interface

**FIGURE 7:**
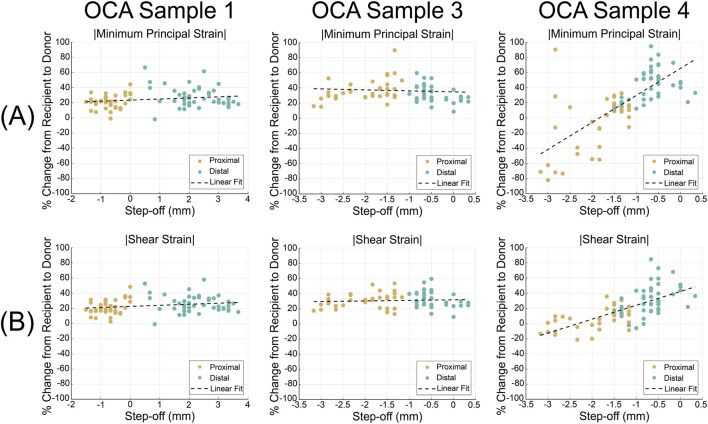
Visualization of the relationship between graft step-off distance and the percent change in strain between recipient and donor cartilage for each OCA sample. Scatter plots show the percent change in the magnitude of minimum principal strain ( $E_{\min}$ ) **(A)** and maximum shear strain ( $E_{\max\;shear}$ ) **(B)** from the recipient to the donor cartilage as a function of local cartilage step-off distance for OCA Samples 1, 3, and 4. Step-off distance was measured in each slice of the 1–2 plane as the difference between the start of the recipient and donor subchondral bone surfaces at the graft interface, with negative values indicating recessed donor cartilage and positive values indicating elevated donor cartilage. Points from the proximal and distal sides of the graft are shown separately. Dashed lines illustrate simple linear fits included for visualization only.

To visualize how the geometric mismatch between the donor and recipient may be related to local mechanical behavior after OCA transplantation, the percentage change in strain outcome for $E_{\min}$ and $E_{\max\;shear}$ from the recipient to the donor cartilage was plotted against the local step-off distance for each OCA sample (Figure 7). Due to the small sample size and the single donor for all OCA procedures, no formal statistical analyses were performed on the data.

Across all samples, step-off values ranged from recessed (up to −3.2 mm) to elevated (up to 3.6 mm), and the percentage changes for both strain outcomes varied across this range. For all samples, the recessed donor cartilage tended to be on the proximal side, while the elevated cartilage was on the distal side. OCA sample 4 displayed a clear directional pattern where a more negative step-off distance tended to correspond to a negative percentage change in strain, especially in $E_{\min}$ . This indicated that there were higher strain magnitudes in the adjacent recipient cartilage compared to the recessed donor cartilage. OCA samples 1 and 3 showed no direct trends with the strain change and the step-off distance, but the donor cartilage consistently had a slightly higher magnitude than the recipient at 20%–40% for $E_{\min}$ (Figure 7A), and 20%–30% in $E_{\max\;shear}$ (Figure 7B).

Overall, these exploratory observations suggest the geometric mismatch at the graft-host interface may influence the local strain difference in some of the samples. However, this step-off distance alone does not fully account for the strain variation since there may be additional mechanical, geometric, and biological factors not captured in this study.

## 4 Discussion

Understanding the changes in compression mechanics of the patellar articular cartilage following OCA transplantation is crucial to predict its short and long-term survivability. Measuring full volume displacement and strain maps in the patella in both intact and OCA-treated configurations could inform how localized changes may influence graft integration, chondrocyte viability, and the long-term durability of the repair. This is the first study to use displacement-encoded MRI to obtain full-volume, three-dimensional maps of intratissue displacement and strain in human patellar articular cartilage before and after osteochondral allograft transplantation under controlled indentation. Overall, global displacement patterns in OCA-treated samples resembled those of intact samples, while localized alterations in strain distribution were observed around portions of the graft interface in some samples. Bands of elevated minimum principal and shear strain were present in certain OCA-treated samples, but were not uniformly observed across all samples.

Exploratory plots of step-off distance versus strain change (Figure 7) showed sample-specific patterns in how geometric mismatch may influence mechanical behavior after transplantation. Notably, OCA Sample 4 displayed directional trends between geometric mismatch and donor-recipient strain differences, where larger negative step-off values (indicating recessed donor cartilage) coincided with a more negative percentage change in strain. This reflects higher strain magnitudes in the recipient cartilage than in the donor cartilage. This sample-specific pattern is consistent with HSR predicted in FE simulations under larger donor-recipient thickness mismatches (Rosario et al., 2023). However, these patterns were not consistent across all samples, which highlights the need for cautious interpretation. OCA Samples 1 and 3 exhibited more variable trends with modest donor-recipient strain differences across the step-off range, likely due to the recipient being farther away from the indentation location. Taken together, these exploratory observations indicate that while step-off distance may contribute to local strain variation, additional mechanical and biological factors likely affect donor-recipient cartilage mechanics, which may ultimately compromise graft integration and long-term survivability.

The observed reduction in force, particularly at the 0.5 mm compression following the OCA procedure, suggests a decrease in apparent stiffness of the cartilage in the indentation location or increased local compliance at the graft site. The absence of prominent HSRs may reflect the sub-physiological magnitude of compression ( ≤ 120 N) which fall below patellofemoral joint forces during walking or running (0.9–5.2× BW) (Hart et al., 2022; Suzuki et al., 2012; Wang et al., 2023). For our samples, the mean BW is 60.2 ± 6.9 kg, or ∼591 N. This means that the patella would experience ∼532 N during walking. These loads may have failed to trigger the high shear or compressive strains between the donor and recipient cartilage compression that we previously predicted in our FE models (Rosario et al., 2023). The indentations performed on the cartilage produced spherical through-thickness displacement contours in the loading direction and lateral displacement of the cartilage as it bulged during compression. This method lacked the complex flexion-dependent stress distribution seen *in vivo* (Escamilla et al., 2009; Huberti and Hayes, 1984). For both set of samples, intact and OCA transplanted, strain fields were located under the indenter with the highest magnitude of the minimum principal strain occurring at the indentation site. Importantly, Sample 1 displayed higher strain intensities than the other intact samples in both the full volume maps and the 95th-percentile projections (Figures 5, 6). Although the mean value for the indentation force for the 2 mm (effective displacement of 0.5 mm) is reported in Table 1, the individual force observed for Sample 1 was the highest of all the samples at 150 N. This likely contributed to the elevated strain magnitude observed, especially when compared to the other intact samples and OCA-treated samples. It is also important to highlight that despite the lower forces observed in the OCA-treated samples (Table 1), strain values under the indenter and near the graft rim achieved strain values up to 0.15. Local elevated strain bands were observed in two of the three OCA samples which may suggest early signature of high strain concentration. These strains bands may represent the beginning of HSR predicted in FE models (Rosario et al., 2023), which would be more pronounced during physiologic conditions.

The lack of consistent HSR in our tested protocol highlights the sensitivity of cartilage mechanics to the loading environment. *In vivo*, PFJ contact is characterized by variable force vectors depending on knee flexion, patellar tilt, and maltracking (Suzuki et al., 2012; Besier et al., 2005). Furthermore, our *ex vivo* conditions did not replicate osteochondral allografts with osseous integration or biological remodeling. Additionally, the MRI voxel size used in this study was much larger than the FE mesh element size from computer simulations (Rosario et al., 2023). Taken together, these factors suggest that the absence of HSRs in our data does not rule out their presence under more physiological or long-term loading conditions.

Most prior work examining patellar cartilage biomechanics has relied on indirect or surface level measures. MRI-based deformation studies in cadaveric PFJ reported cartilage thickness changes under static loading, demonstrating that the patella cartilage deforms substantially during physiological forces (Herberhold et al., 1998; 1999). Other work has focused on contact pressure in relation to patellar misalignment or tibial tubercle transfer (Kuroda et al., 2001; Freedman et al., 2015), and MRI-based techniques have also been applied to quantify cartilage deformation in the tibiofemoral joint (Chan et al., 2016; Lee et al., 2023); however, none of these studies have directly addressed the mechanics of the cartilage in the PFJ. Recent work using X-ray micro-CT combined with digital volume correlation has produced full-field three-dimensional strain maps in small-animal osteochondral plugs (Davis et al., 2025), demonstrating the utility of various approaches to obtain volumetric strain for osteochondral tissues. Research on patellofemoral OCA procedures has primarily centered on clinical outcomes or computational simulations. For example, clinical studies have demonstrated satisfactory graft survivorship at 5–10 years but increased reoperation and failure rates at 15 years (Familiari et al., 2018; Chahla et al., 2019). Our experimental approach directly bridges these fields by providing a method to validate FE model predictions and contextualizing clinical failure by providing the first quantification of full volume, three-dimensional strain distributions in patellar cartilage before and after OCA transplantation. While displacement-encoded MRI has been successfully applied to tibiofemoral cartilage *in vivo* and in *ex vivo* explants (Lee et al., 2023; Miller et al., 2025; Neu and Walton, 2008), it has not previously been extended to the PFJ or to OCA transplantation. By establishing that this imaging approach can capture full volume patellar cartilage strains, our study lays the groundwork for translational applications that link computational predictions with clinical outcomes.

This work has several limitations. First, the sample size was small (n = 4), and all three OCA transplant recipients received grafts from a single donor patella, which limits statistical independence and reduces generalizability across samples. For this reason, the findings should be interpreted descriptively rather than inferentially. Second, the use of frozen cadaveric tissue may have compromised the biomechanical response and hydration of the cartilage compared to fresh or *in vivo* cartilage. Third, our loading protocol involved sub-physiological forces and centralized indentation rather than physiological trochlear-patellar contact across varying angles. Although indentation allowed us to consistently apply the same compression to all samples, prior work has demonstrated that PFJ contact mechanics are highly flexion-dependent, with peak pressure occurring between 60°–90° of flexion (Escamilla et al., 2009; Huberti and Hayes, 1984), which would require a modification of the boundary conditions. Under these loads, HSR at the donor-recipient interface may not fully manifest. Fourth, the displacement-encoded MRI acquisition used a single encoding wavelength and anisotropic voxel dimensions (1.5–2.0 mm in two axes), which required spatial smoothing during processing and likely reduced our ability to resolve steep strain gradients, particularly near the rim of the graft where the cartilage mechanics are most sensitive. Because the FE mesh used was finer than the voxel resolution in the longitudinal and transverse direction, strain components involving the 2-direction (like shear) may be attenuated or oversmoothed and should be interpreted with caution. Finally, while our exploratory step-off distance plots illustrated qualitative, sample-specific trends between geometric mismatch and donor-recipient strain differences, these patterns were not consistent across samples and were intended only as descriptive visualizations. This further supports that step-off distance alone cannot fully explain the local strain variation in the graft interface in our samples and that additional unmeasured mechanical and biological factors contribute to strain behavior following OCA transplantation.

In future work we will address these limitations by using fresh cadaveric samples and testing within a window of time in which chondrocyte viability can be assured. This will also expand the tissue cohorts to multiple recipients and donors, which will allow us to quantify cartilage thickness mismatches and perform relevant statistical analyses. We also plan on applying physiological PFJ loading considering a variety of knee flexion angles and reducing the compliance of the current system. Additionally, we plan to use inverse methods such as variational system identification with partial differential equation optimization (Wang et al., 2021) for constitutive parameter inference of the patella articular cartilage, incorporating full volume displacement data. Developing a constitutive model from these datasets will allow us to replicate these results in a FE environment and accurately parameterize different factors like the cartilage thickness, loading conditions, and boundary conditions on patient-specific models.

These findings underscore the importance of achieving cartilage-level congruity during patellar OCA transplantation. In some samples, modest step-off distances coincided with localized changes in the strain, suggesting that geometric mismatch may influence the strain distribution in certain contexts, although this pattern was not consistent across all samples. Because elevated shear and compressive strains are linked to chondrocyte death and early matrix disruption (Kosonen et al., 2023; Bonnevie et al., 2018), the combination of displacement-encoded MRI and FE constitutive modeling offers a promising framework to detect unfavorable graft environments. Incorporating this framework into preclinical evaluation could inform graft selection and improve long-term outcomes for patients undergoing OCA transplantation.

In summary, we present the first experimental study that provides full volume displacement and strain maps of intact and OCA-transplanted human patella cartilage in controlled compression using a modified displacement-encoded MRI technique (APGSTEi). While OCA transplantation did not produce uniform changes across samples, two of the three OCA-treated samples exhibited localized alterations in strain distribution near the graft rim compared with their intact states. These strain features occurred despite the lower average indentation forces at each displacement relative to the intact condition. Exploratory observations of step-off distances indicated that cartilage-level incongruity may correspond with localized strain differences in certain samples, although this relationship was not consistent across all samples. Altogether, these findings provide an initial experimental foundation for evaluating how geometric and mechanical factors shape the local mechanical environment following patellar OCA transplantation and provide a basis for further studies investigating how imaging, modeling, and surgical techniques might influence long-term clinical outcomes.

## Data Availability

The raw data supporting the conclusions of this article will be made available by the authors, without undue reservation.
